# Five new cryptic freshwater gastropod species from New Caledonia (Caenogastropoda, Truncatelloidea, Tateidae)

**DOI:** 10.3897/zookeys.523.6066

**Published:** 2015-09-28

**Authors:** Martin Haase, Susan Zielske

**Affiliations:** 1 Vogelwarte, Zoologisches Institut und Museum, Ernst-Moritz-Arndt-Universität Greifswald, Soldmannstraße 23, D-17489 Greifswald, Germany

**Keywords:** Conservation, cryptic species, endemic, integrative taxonomy, IUCN, New Caledonia, South Pacific, spring snails, Tateidae

## Abstract

During the course of a project aiming at the reconstruction of the colonization of the South Pacific islands by tateid gastropods based on molecular data we discovered five new species on New Caledonia belonging to the genera *Hemistomia* and *Leiorhagium*, respectively. We describe these species based on morphological, anatomical and genetic data. All five species are morphologically cryptic as they closely resemble or are even indistinguishable from known species stressing the importance of a comprehensive taxonomic approach integrating several methods. As a consequence of their small and fragmented geographic ranges and the rapidly progressing anthropogenic land cover changes on New Caledonia, all five species qualify as critically endangered according to the criteria of the IUCN.

## Introduction

**Figure 1.**
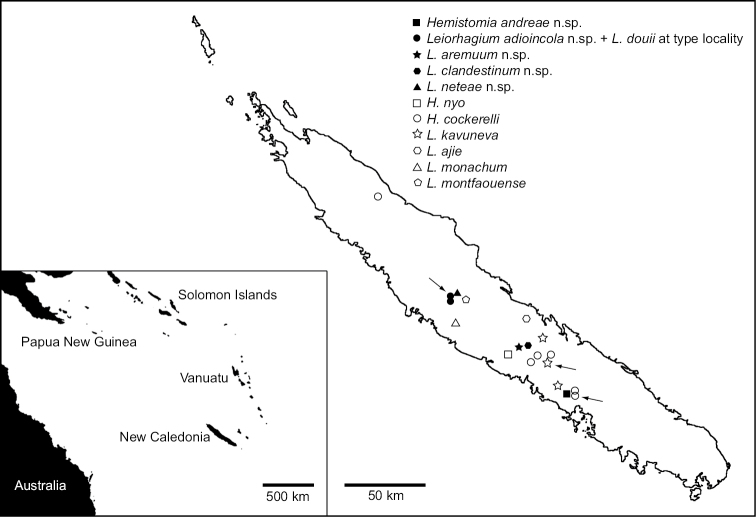
Localities of new species and samples used for morphometric comparisons. Inset shows position of New Caledonia in the Southwest Pacific. Arrows indicate type localities of species represented by more than one sample (see also Table 1).

**Figure 2.**
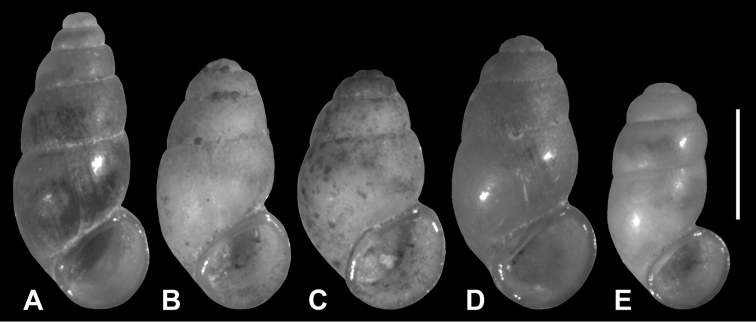
Holotypes. **A** *Hemistomia andreae* sp. n. **B** *Leiorhagium adioincola* sp. n. **C** *Leiorhagium aremuum* sp. n. **D** *Leiorhagium clandestinum* sp. n. **E** *Leiorhagium neteae* sp. n. Scale bar = 1 mm.

New Caledonia is famous for being a biodiversity hotspot harboring a high number of endemic species (Myers et al. 2000) including a radiation of small freshwater gastropods belonging to the family Tateidae. This radiation is probably of Oligocene origin and comprises more than 50 species in seven genera (Haase and Bouchet 1998, Zielske and Haase 2015). Many of these species are extreme narrow-range endemics known from only few or single sites (Haase and Bouchet 1998), a pattern typical for Truncatelloidea in freshwaters worldwide (e.g. Giusti and Pezzoli 1980, Radoman 1983, Haase 1996, 2008, Ponder and Colgan 2002, Liu and Hershler 2005, Hershler et al. 2011, Delicado and Ramos 2012). In the frame of a project aiming at the reconstruction of the colonization of the South Pacific islands by tateids based on molecular data (Zielske and Haase 2014a, b, 2015, Zielske, Ponder and Haase in preparation) we visited New Caledonia in May 2012 in order to collect suitable material for sequencing. During this expedition we found five new species of the genera *Hemistomia* Crosse, 1872 and *Leiorhagium* Haase & Bouchet, 1998, respectively (Figs 1, 2), which we describe herein based on morphological, anatomical and genetic data. All five species qualify as morphologically cryptic as they closely resemble or are even indistinguishable from known species (see Pfenninger and Schwenk 2007). The discovery of new cryptic species was predicted by Haase and Bouchet (1998), whose revision was based solely on morphology and anatomy. In general, cryptic species are common among different spring snail families of Truncatelloidea (e.g., Liu et al. 2003; Haase et al. 2007; Delicado and Ramos 2012; Collado et al. 2013).

**Table 1.** Locality data of all samples and GenBank accession numbers of specimens represented in phylogeny (see also Fig. 1). The last three species represent the outgroup. Specimens are only distinguished in two cases where more than 1 sequence per sample was used. For museum catalog numbers of NeCa-sample voucher material see Zielske and Haase (2015). Paratypes of species described by Haase and Bouchet (1998) used in morphometric comparisons are accompanied by catalog numbers from the museum in Paris, because these have been assigned only recently.

Species, sample	Locality	Latitutde, longitude	COI	16S	IT2
*Hemistomia andreae*, NeCa 12_1 *Hemistomia andreae*, NeCa 12_2	Bouloupari, Ouaméni valley	21°49'46.9"S; 165°56'42.9"E	KJ490851 KJ490852	KJ490767 ---	KJ490691 ---
*Hemistomia cockerelli*, paratypes MNHN IM-2012-2694	Bouloupari, Ouaméni, prop. Debels	21°49'12.0"S; 166°56'36.0"E			
*Hemistomia cockerelli*, NeCa 11	Bouloupari, Ouitchambo	21°48'16.8"S; 166°00'00.8"E			
*Hemistomia cockerelli*, NeCa 17	Moindou, road toward barrage	21°39'52.8"S; 165°43"10.3"E	KJ490857	KJ490772	KJ490696
*Hemistomia cockerelli*, NeCa 21A	Farino, Sentier de la Cascade et des Sources	21°38'11.9"S; 165°46'36.6"E	KJ490863	---	KJ490702
*Hemistomia cockerelli*, NeCa 36	Sarraméa, track to “Trou d’Eau”	21°38'22.1"S; 165°51'37.5"E			
*Hemistomia cockerelli*, NeCa 54	Hienghène, Tendo	20°42'54.7"S; 164°49'20.8"E			
*Hemistomia eclima*, NeCa 19	Moindou, road toward barrage	21°39'58.4"S; 165°43'08.2"E	KJ490858	KJ490773	KJ490697
*Hemistomia fabrorum*, NeCa 1	Dumbéa, Koé, prop. Oesterlin	22°08'59.0"S; 166°29'10.6"E	KJ490829	KJ490749	KJ490670
*Hemistomia fabrorum*, NeCa 25B	Sarraméa, road side of RPN 5	21°34'15.7"S; 165°49'41.2"E	KJ490867	KJ490781	KJ490704
*Hemistomia minor*, NeCa 30	Moindou, road side SW Katrikoin	21°34'21.6"S; 165°41'02.5"E	KJ490872	KJ490786	KJ490709
*Hemistomia nyo*, NeCa 35	Bourail, Oua Oué	21°36'50.3"S; 165°35'31.5"E	KJ490880	KJ490791	KJ490716
*Hemistomia oxychila*, NeCa 43A	Poya, road side between Nétéa and Goipin	21°16'06.0"S; 165°14'32.0"E	KJ490893	KJ490804	KJ490726
*Hemistomia rusticorum*, NeCa 6A	Bouloupari, road side N Nassirah	21°48'08.0"S; 166°04'14.6"E	KJ490836	KJ490755	KJ490677
*Hemistomia winstonefi*, NeCa 3B	Mont Dore, Rue des Roseaux, prop. Solier	22°15'42.4"S; 166°34'08.7"E	KJ490834	KJ490753	KJ490675
*Leiorhagium adioincola*, NeCa 43B	Poya, side of road to Goipin	21°16'06.0"S; 165°14'32.0"E	KJ490895	KJ490806	KJ490728
*Leiorhagium adioincola*, NeCa 49	Poya, stream into Grotte d‘Adio	21°15'24.4"S; 165°14'46.4"E	KJ490901	KJ490812	KJ490734
*Leiorhagium ajie*, paratypes MNHN IM-2012-2688	Houailou, Néoua	21°24'00.0"S; 165°38'54.0"E			
*Leiorhagium aremuum*, NeCa 33_1 *Leiorhagium aremuum*, NeCa 33_2	Moindou, Aremu valley	21°35'04.8"S; 165°39'07.5"E	KJ490878 KJ490879	KJ490789 KJ490790	KJ490714 KJ490715
*Leiorhagium clandestinum*, NeCa 30B	Moindou, road side SW Katrikoin	21°34'21.6"S; 165°41'02.5"E	KJ490874	---	KJ490711
*Leiorhagium douii*, paratypes MNHN IM-2012-2681	Poya, Grotte d’Adio	21°15'30.0"S; 165°14'30.0"E			
*Leiorhagium inplicatum*, NeCa 9B	Bouloupari, road side of RP 4	21°44'30.9"S; 166°05'57.9"E	KJ490845	KJ490762	KJ490685
*Leiorhagium kavuneva*, paratypes MNHN IM-2012-2690	Sarraméa, prop. Bonnard	21°39'00.0"S; 165°50'48.0"E			
*Leiorhagium kavuneva*, NeCa 15B	Bouloupari, Oua Tom	21°47'24.4"S; 165°54'51.2"E	KJ490855	KJ490770	KJ490694
*Leiorhagium kavuneva*, NeCa 27	Kouaoua, road side N Koh	21°30'52.2"S; 165°48'05.0"E	KJ490869	KJ490783	KJ490706
*Leiorhagium kavuneva*, NeCa 29	Kouaoua, road side N Koh	21°32'02.6"S; 165°49'27.2"E			
*Leiorhagium monachum*, paratypes MNHN IM-2012-2679	Poya, Mt. Krapé	21°23'12.0"S; 165°14'30.0"E			
*Leiorhagium montfaouense*, paratypes MNHN IM-2012-2684	Poya, Montfaoué	21°16'48.0"S; 165°17'42.0"E			
*Leiorhagium neteae*, NeCa 44B	Poya, beginning of road into Vallée d’Adio	21°14'47.9"S; 165°15'45.0"E	KJ490897	KJ490808	KJ490730
*Leiorhagium orokau*, NeCa 42	Poya, near Nétéa	21°16'32.2"S; 165°12'17.6"E	KJ490891	KJ490802	KJ490724
*Leiorhagium orokau*, NeCa 57	Hienghène, Tendo	20°42'43.9"S; 164°47'47.5"E	KJ490912	KJ490823	KJ490744
*Crosseana crosseana*, NeCa 51	Koumac, seepage in N of town	20°32'32.2"S; 164°18'33.0"E	KJ490904	KJ490815	KJ490737
*Crosseana melanosoma*, NeCa 50	Voh, Boyen, overflow of reservoir	20°49'13.6"S; 164°36'56.4"E	KJ490902	KJ490813	KJ490735
*Kanakyella gentilsiana*, NeCa 58	Hienghène, Tendo	20°42'22.4"S; 164°47'20.0"E	KJ490914	KJ490825	KJ490746

## Material and methods

Snails were fixed in 70% ethanol in the field, transferred to propylene glycol for shipping by courier, and returned to ethanol, this time 96%, after arrival in our lab. For measurements, up to 20 snails per sample were photographed under a Zeiss SteREO Discovery.V20 dissecting microscope with a Zeiss Axio Cam MR3. Five dimensions – shell height, shell width, aperture height, aperture width, body whorl width – were measured using the program AxioVision 40 V4.8. (Zeiss) and whorls counted to the nearest eighth (Kerney and Cameron 1979). Up to six shells were dissolved in diluted hydrochloric acid for dissections. Anatomies were photographed as well. These digital images served as template for drawings made on a graphical tablet. For scanning electron microscopy up to three shells, radulae and opercula were cleaned in 5% sodium hypochlorite. The cephalopodia of up to two males were dried using hexamethyldisilazane (Nation 1983). After sputter coating with gold objects were investigated in a Zeiss EVO LS10 Scanning Microscope.

Morphometric analyses of shell measurements including canonical variates analyses (CVA) maximizing the differentiation of groups in multivariate space, multivariate analyses of variance (MANOVA), assignment tests, and Hotelling’s T^2^-tests were conducted in PAST 2.12 (Hammer et al. 2001). Sequential Bonferroni-correction was applied to multiple tests. These analyses also included samples of known, similar species the new ones could be mistaken for (Table 1). The selection of species used in comparisons was based on the phylogenetic analysis.

Phylogenetic analyses were based on a selection of sequences generated by Zielske and Haase (2015), who analyzed fragments of the mitochondrial genes cytochrome oxidase subunit I (COI) and 16S rRNA as well as the nuclear internal transcribed spacer 2 (ITS2). For lab protocols see Zielske and Haase (2014a, 2015). We restricted the analysis to 3 specimens per species at most and used *Kanakyella gentilsiana*, *Crosseana crosseana*, and *Crosseana melanosoma* as outgroups (Table 1). The alignment of 16S rRNA and ITS2 was generated using secondary structure information using RNAsalsa 0.8.1 (Stocsits et al. 2009) (for details see Zielske and Haase 2015) and checked for ambiguous and randomly similar sites in Aliscore 2.0 (Misof and Misof 2009). We defined seven partitions. PartitionFinder 1.1.0 (Lanfear et al. 2012) identified the following scheme and substitution models as optimal among all possible combinations of separate and merged partitions: COI 1^st^ positions (TrNef+I), COI 2^nd^ positions (F81), COI 3^rd^ positions (TVM+I+Γ), 16S rRNA loops (TrN+ Γ), ITS2 loops (TrNef+I+Γ), joint stems of 16S rRNA and ITS2 (K80+I). With these settings, tree reconstructions were conducted in a maximum likelihood (ML) framework using GARLI 2.01 (Zwickl 2006) with 500 replicates. Robustness was assessed by bootstrapping with 200 replicates.

Type and non-type material is deposited at the Museum National d’Histoire Naturelle in Paris (MNHN) and at the Naturhistorisches Museum Wien (NHMW).

## Results

### Systematic descriptions

Diagnoses and descriptions of *Hemistomia* and *Leiorhagium* and data used in our comparisons with the new species were provided by Haase and Bouchet (1998). Locality data include site number, district capital, site, coordinates, and date of collection. Shell measurements are given in Table 2 and not repeated in the descriptions.

**Table 2.** Morphometry. Measurements in mm. Shell measures: AH, aperture hight; AW, aperture width; BWW, width of body whorl; SH, shell height; SW, shell width; W, number of whorls; statistics: CV, coefficient of variation corrected for unequal sample sizes; max, maximum; min, minimum; SD, standard deviation. First line of new species contains measurements of holotypes. Note that the holotype was only in case of *Leiorhagium clandestinum* included in the descriptive statistics. Numbers of whorls were only counted in the new species as this parameter was not used in the statistical analyses.

**New species**							
	SH	SW	AH	AW	BWW	SH/SW	W
*Hemistomia andreae* sp. n. (*N*=20)	2.70	1.25	0.90	0.87	1.08	2.17	5.4
min	2.40	1.10	0.80	0.75	0.97	2.00	4.50
max	2.78	1.28	0.93	0.91	1.08	2.35	5.50
mean	2.60	1.18	0.85	0.82	1.02	2.20	5.14
median	2.60	1.17	0.85	0.82	1.01	2.23	5.25
SD	0.11	0.05	0.04	0.04	0.03	0.11	0.28
CV	4.40	3.93	4.23	4.54	2.77	4.94	5.49
*Leiorhagium adioincola* sp. n. NeCa 49 (*N*=20)	2.29	1.24	0.88	0.87	1.09	1.84	4.50
min	2.10	1.16	0.83	0.83	1.04	1.71	4.13
max	2.42	1.31	0.96	0.96	1.15	1.90	4.75
mean	2.25	1.25	0.88	0.89	1.10	1.80	4.36
median	2.24	1.24	0.88	0.89	1.10	1.80	4.25
SD	0.09	0.04	0.04	0.03	0.03	0.05	0.18
CV	4.21	2.96	4.15	3.92	2.94	2.91	4.24
*Leiorhagium aremuum* sp. n. (*N*=20)	2.19	1.35	0.97	0.91	1.16	1.62	4.25
min	2.03	1.29	0.87	0.86	1.10	1.53	3.75
max	2.43	1.46	1.03	1.00	1.25	1.69	4.25
mean	2.19	1.35	0.94	0.92	1.16	1.62	4.03
median	2.15	1.35	0.93	0.92	1.17	1.62	4.00
SD	0.11	0.05	0.04	0.04	0.04	0.04	0.15
CV	4.92	4.06	4.76	4.54	3.77	2.71	3.78
*Leiorhagium clandestinum* sp. n. (*N*=4)	2.49	1.32	0.94	0.95	1.16	1.91	4.50
min	2.23	1.26	0.89	0.88	1.07	1.77	4.25
max	2.49	1.32	0.94	0.95	1.16	1.91	4.50
mean	2.38	1.28	0.91	0.92	1.10	1.86	4.41
median	2.41	1.27	0.90	0.93	1.09	1.89	4.44
SD	0.11	0.03	0.02	0.03	0.04	0.06	0.12
CV	4.83	2.30	2.68	3.44	4.00	3.52	2.89
*Leiorhagium neteae* n. sp. (*N*=18)	2.07	1.12	0.75	0.77	0.91	1.84	4.50
min	1.85	0.97	0.65	0.70	0.82	1.76	4.25
max	2.23	1.17	0.79	0.80	0.95	2.01	5.00
mean	2.05	1.09	0.73	0.75	0.88	1.88	4.46
median	2.04	1.10	0.73	0.75	0.87	1.88	4.38
SD	0.12	0.05	0.03	0.03	0.03	0.07	0.19
CV	6.05	4.82	4.71	4.69	3.99	3.72	4.25
**Material for comparisons**							
	SH	SW	AH	AW	BWW	SH/SW	
*Hemistomia cockerelli* Types (*N*=20)							
min	2.58	1.18	0.88	0.83	1.03	2.05	
max	3.21	1.39	1.03	0.97	1.16	2.40	
mean	2.79	1.27	0.94	0.91	1.09	2.19	
median	2.74	1.25	0.93	0.90	1.09	2.18	
SD	0.17	0.06	0.04	0.04	0.05	0.09	
CV	6.20	4.91	4.31	4.52	4.23	4.36	
*Hemistomia cockerelli* NeCa11 (*N*=20)							
min	2.20	1.06	0.77	0.73	0.94	1.93	
max	2.48	1.25	0.87	0.91	1.04	2.28	
mean	2.33	1.13	0.81	0.81	0.97	2.06	
median	2.32	1.12	0.80	0.80	0.96	2.03	
SD	0.08	0.04	0.03	0.04	0.02	0.10	
CV	3.49	3.70	3.36	4.60	2.36	4.75	
*Hemistomia cockerelli* NeCa17 (*N*=20)							
min	2.35	1.16	0.83	0.83	1.04	1.96	
max	2.62	1.28	0.92	0.93	1.14	2.19	
mean	2.50	1.21	0.87	0.87	1.08	2.07	
median	2.51	1.21	0.87	0.88	1.08	2.07	
SD	0.07	0.04	0.03	0.03	0.02	0.07	
CV	2.90	3.20	3.16	3.25	2.32	3.43	
*Hemistomia cockerelli* NeCa21A (*N*=8)							
min	2.26	1.09	0.74	0.77	0.96	2.03	
max	2.74	1.23	0.89	0.87	1.08	2.38	
mean	2.49	1.17	0.84	0.83	1.03	2.12	
median	2.49	1.17	0.85	0.83	1.05	2.09	
SD	0.14	0.05	0.04	0.03	0.04	0.11	
CV	5.87	4.33	5.41	3.89	3.66	5.50	
*Hemistomia cockerelli* NeCa36 (*N*=13)							
min	2.32	1.14	0.79	0.82	1.03	1.97	
max	2.64	1.23	0.91	0.91	1.12	2.14	
mean	2.43	1.18	0.85	0.85	1.06	2.05	
median	2.42	1.19	0.86	0.85	1.06	2.04	
SD	0.10	0.03	0.03	0.03	0.03	0.05	
CV	4.25	2.42	3.79	3.22	2.64	2.64	
*Hemistomia cockerelli* NeCa54 (*N*=20)							
min	2.28	1.16	0.78	0.82	1.04	1.86	
max	2.63	1.31	0.96	0.93	1.14	2.14	
mean	2.47	1.23	0.87	0.88	1.09	2.00	
median	2.47	1.23	0.86	0.87	1.10	2.02	
SD	0.10	0.04	0.04	0.03	0.03	0.07	
CV	4.18	3.08	4.18	3.28	2.62	3.62	
*Hemistomia nyo* NeCa35 (*N*=7)							
min	2.43	1.25	0.88	0.89	1.09	1.93	
max	2.75	1.34	0.96	0.96	1.15	2.08	
mean	2.62	1.30	0.92	0.92	1.12	2.01	
median	2.69	1.30	0.91	0.91	1.11	2.03	
SD	0.12	0.04	0.03	0.03	0.02	0.06	
CV	4.80	2.84	3.30	3.10	2.00	3.00	
*Leiorhagium ajie* Types (*N*=6)							
min	2.35	1.31	0.93	0.94	1.12	1.61	
max	2.74	1.62	1.10	1.06	1.34	1.80	
mean	2.50	1.46	1.01	1.00	1.25	1.72	
median	2.43	1.46	1.01	1.00	1.27	1.70	
SD	0.16	0.12	0.07	0.05	0.08	0.07	
CV	6.50	8.31	6.95	4.88	6.32	4.12	
*Leiorhagium douii* Types (*N*=20)							
min	1.87	0.98	0.68	0.68	0.86	1.84	
max	2.50	1.16	0.84	0.79	0.97	2.16	
mean	2.06	1.05	0.73	0.71	0.91	1.96	
median	2.02	1.06	0.72	0.71	0.91	1.95	
SD	0.14	0.04	0.04	0.02	0.03	0.08	
CV	7.04	4.02	5.11	3.51	3.21	4.23	
*Leiorhagium kavuneva* Types (*N*=20)							
min	2.17	1.17	0.78	0.82	1.02	1.77	
max	2.42	1.32	0.94	0.93	1.13	1.93	
mean	2.31	1.26	0.88	0.88	1.07	1.84	
median	2.33	1.25	0.89	0.88	1.07	1.85	
SD	0.07	0.04	0.04	0.03	0.03	0.05	
CV	3.24	3.16	4.52	3.15	2.87	2.58	
*Leiorhagium kavuneva* NeCa15B (*N*=20)							
min	2.20	1.21	0.84	0.88	1.07	1.76	
max	2.46	1.31	0.94	0.98	1.20	1.97	
mean	2.34	1.27	0.90	0.92	1.12	1.84	
median	2.35	1.28	0.91	0.92	1.12	1.83	
SD	0.07	0.03	0.03	0.03	0.03	0.06	
CV	3.14	2.30	3.31	3.00	2.54	3.14	
*Leiorhagium kavuneva* NeCa29 (*N*=20)							
min	2.17	1.20	0.85	0.85	1.06	1.76	
max	2.54	1.36	1.00	0.99	1.17	1.97	
mean	2.35	1.28	0.91	0.93	1.12	1.83	
median	2.34	1.27	0.90	0.93	1.13	1.82	
SD	0.10	0.04	0.04	0.04	0.03	0.05	
CV	4.27	3.12	4.50	3.93	2.91	2.62	
*Leiorhagium monachum* Types (*N*=3)							
min	2.07	1.04	0.72	0.69	0.88	1.88	
max	2.18	1.10	0.82	0.78	0.97	2.00	
mean	2.11	1.08	0.76	0.74	0.92	1.96	
median	2.07	1.09	0.76	0.75	0.92	1.99	
SD	0.07	0.03	0.05	0.05	0.05	0.07	
CV	3.36	3.19	7.25	6.89	5.51	3.67	
*Leiorhagium montfaouense* Types (*N*=10)							
min	1.80	1.03	0.68	0.64	0.83	1.76	
max	2.30	1.16	0.81	0.77	0.99	1.99	
mean	2.01	1.08	0.73	0.70	0.90	1.87	
median	2.02	1.05	0.72	0.68	0.89	1.86	
SD	0.15	0.05	0.05	0.04	0.06	0.09	
CV	7.73	4.89	6.40	6.29	6.87	4.79	

#### Genus Hemistomia

Taxon classificationAnimaliaLittorinimorphaTateidae

Crosse, 1872

##### Type species.

*Hemistomia caledonica* Crosse, 1872

#### Hemistomia andreae sp. n.

Taxon classificationAnimaliaLittorinimorphaTateidae

http://zoobank.org/1C80E381-43F7-43EB-9853-425C5C6B925E

##### Type material.

Holotype MNHN IM 2000-27858; paratypes MNHN IM 2000-27859 (> 50), NHMW 110181 (10).

##### Type locality.

NeCa 12, Bouloupari: Ouaméni-valley, small stream on W-side of road in secondary forest, 21°49'46.9"S, 165°56'42.9"E, 22 May 2012.

##### Etymology.

The new species is dedicated to the senior author’s daughter on the occasion of her ‘quinceañera’, the 15^th^ birthday.

##### Diagnosis.

*Hemistomia andreae* sp. n. is very similar to *Hemistomia cockerelli* and *Hemistomia nyo*. It differs from both in a clearer separation of the opercular pegs and a much more delicate penis. The protoconch of the new species has more whorls than *Hemistomia nyo* and the palatal denticle is further behind the outer lip.

##### Shell.

**Figure 3.**
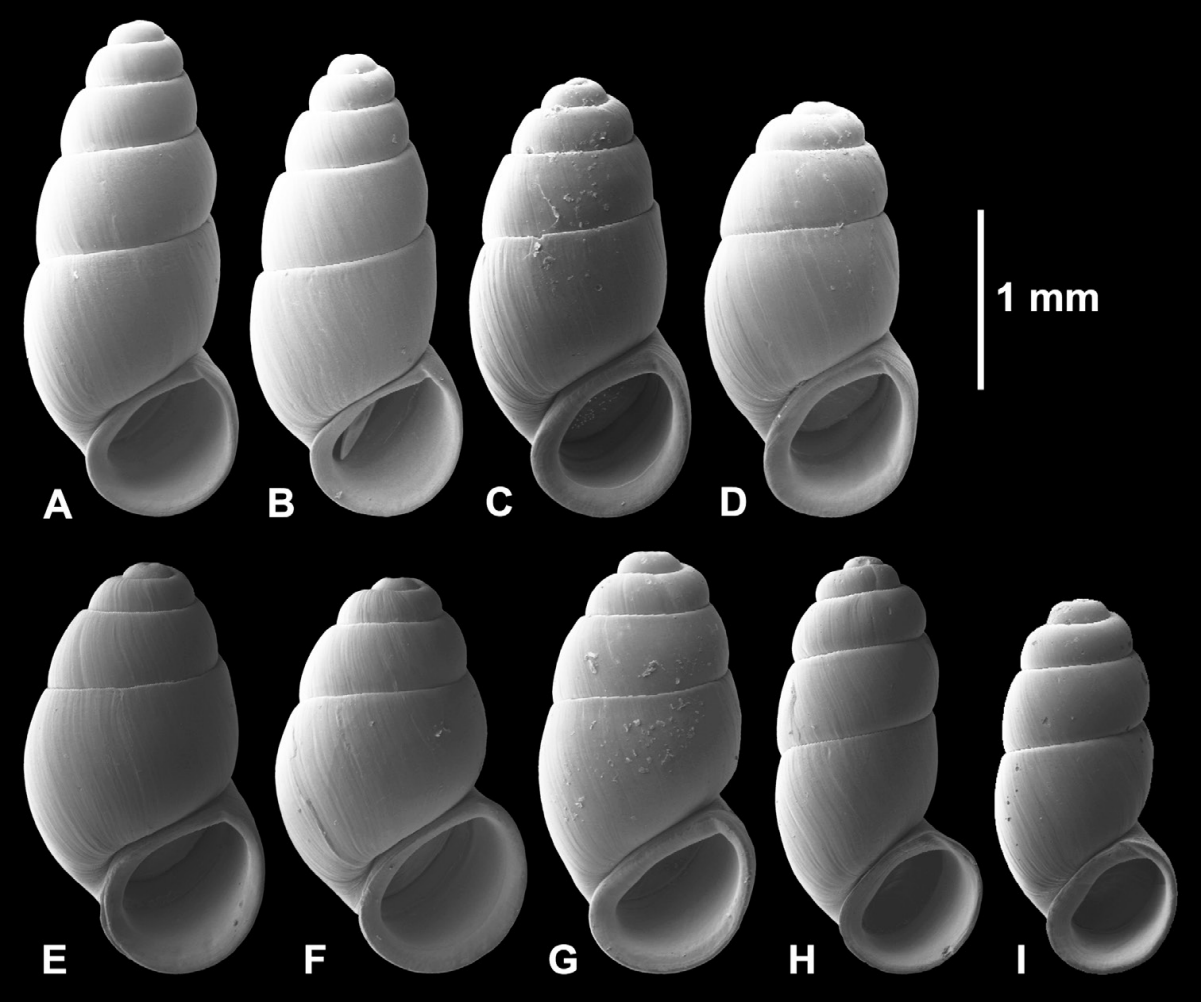
Shells (all paratypes). **A, B** *Hemistomia andreae* sp. n. **C, D** *Leiorhagium adioincola* sp. n. **E, F** *Leiorhagium aremuum* sp. n. **G** *Leiorhagium clandestinum* sp. n. **H, I** *Leiorhagium neteae* sp. n.

**Figure 4.**
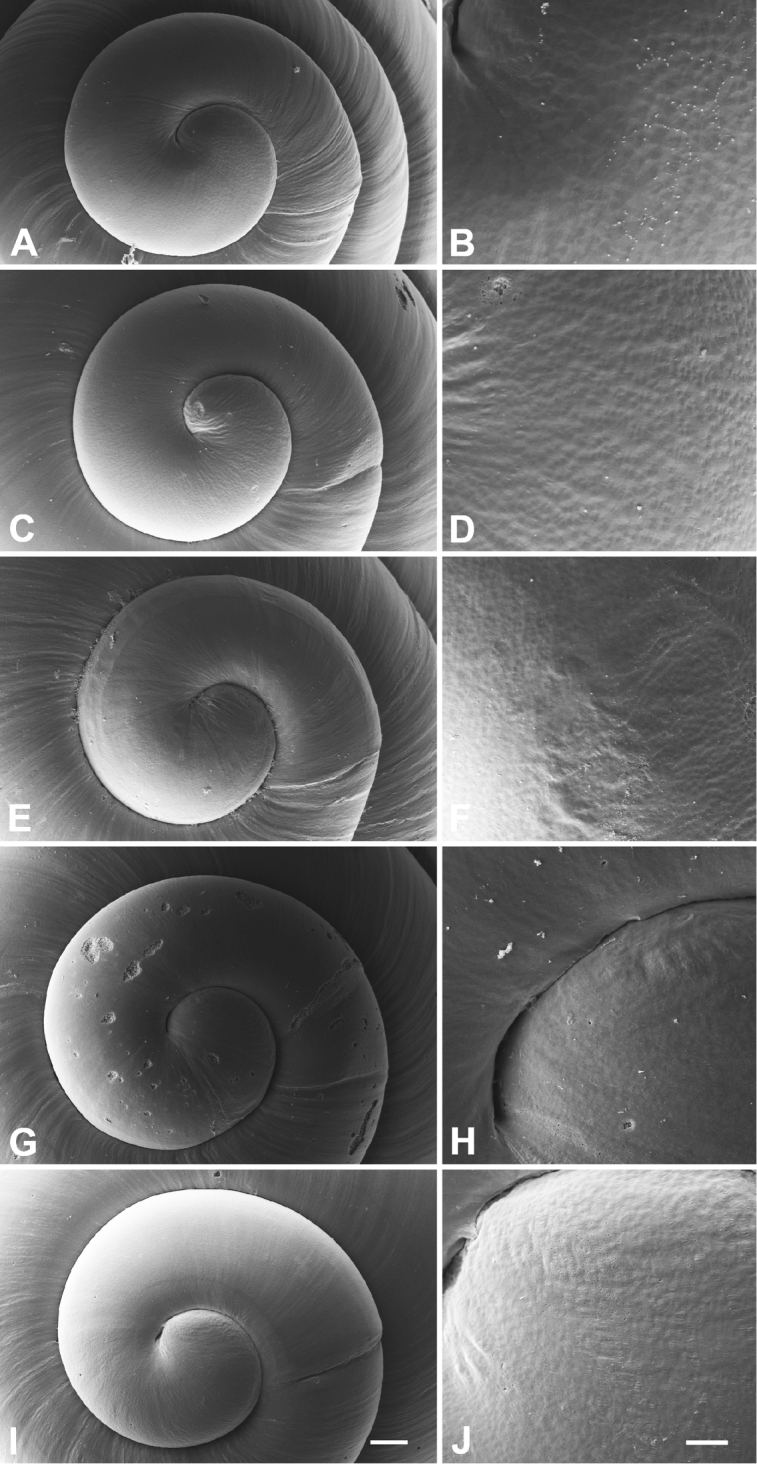
Protoconchs (left) and close-up views of apical microstructure (right). **A, B** *Hemistomia andreae* sp. n. **C, D** *Leiorhagium adioincola* sp. n. **E, F** *Leiorhagium aremuum* sp. n. **G, H** *Leiorhagium clandestinum* **I, J** *Leiorhagium neteae* sp. n. Scale bars 50 µm (**A, C, E, G, I**), 10 µm (**B, D, F, H, J)**.

Conical, 2.2 times higher than wide, 4.5-5.5 whorls, without colour, transparent; protoconch faintly pitted with 1-1.25 whorls; palatal denticle large, elongate, c. 1/3 whorl behind outer lip; with columellar fold in the body whorl; aperture slightly higher than wide (Figs 2A, 3A,B, 4A,B).

##### Operculum.

**Figure 5.**
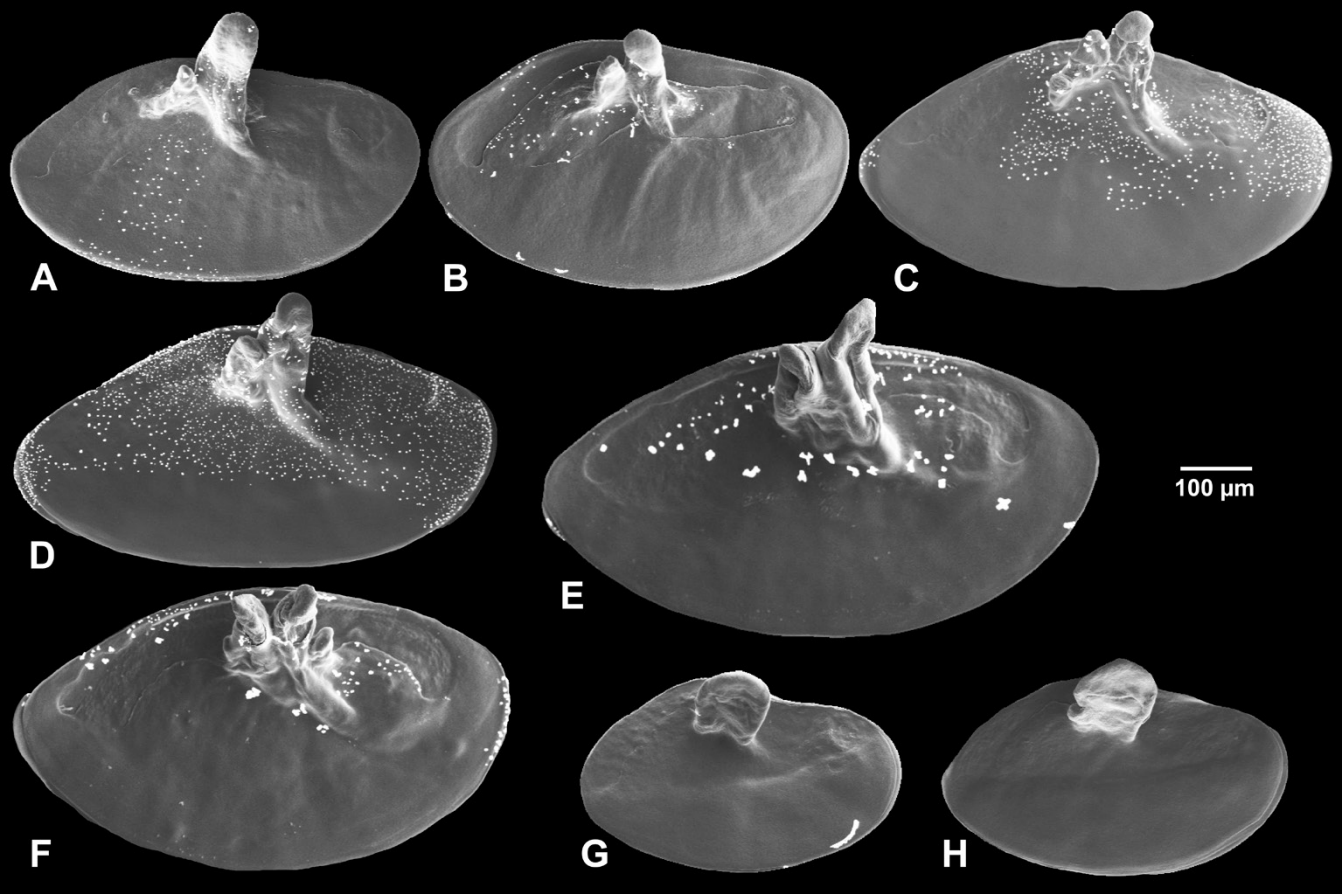
Operculum. **A, B** *Hemistomia andreae* sp. n. **C, D** *Leiorhagium adioincola* sp. n. **E, F** *Leiorhagium aremuum* sp. n. **G, H** *Leiorhagium neteae* sp. n.

Elongate-ellipsoidal, paucisprial, nucleus submarginal, orange, one large and one small non-calcareous white peg, well separated from each other (*N*=5) (Fig. 5A,B).

##### External features.

Epidermis without pigment, eyes black.

##### Mantle cavity.

Ctenidium with 24–26 (2 males) or 25–28 (3 females) filaments; osphradium kidney-shaped, behind middle of ctenidium.

##### Digestive system.

**Figure 6.**
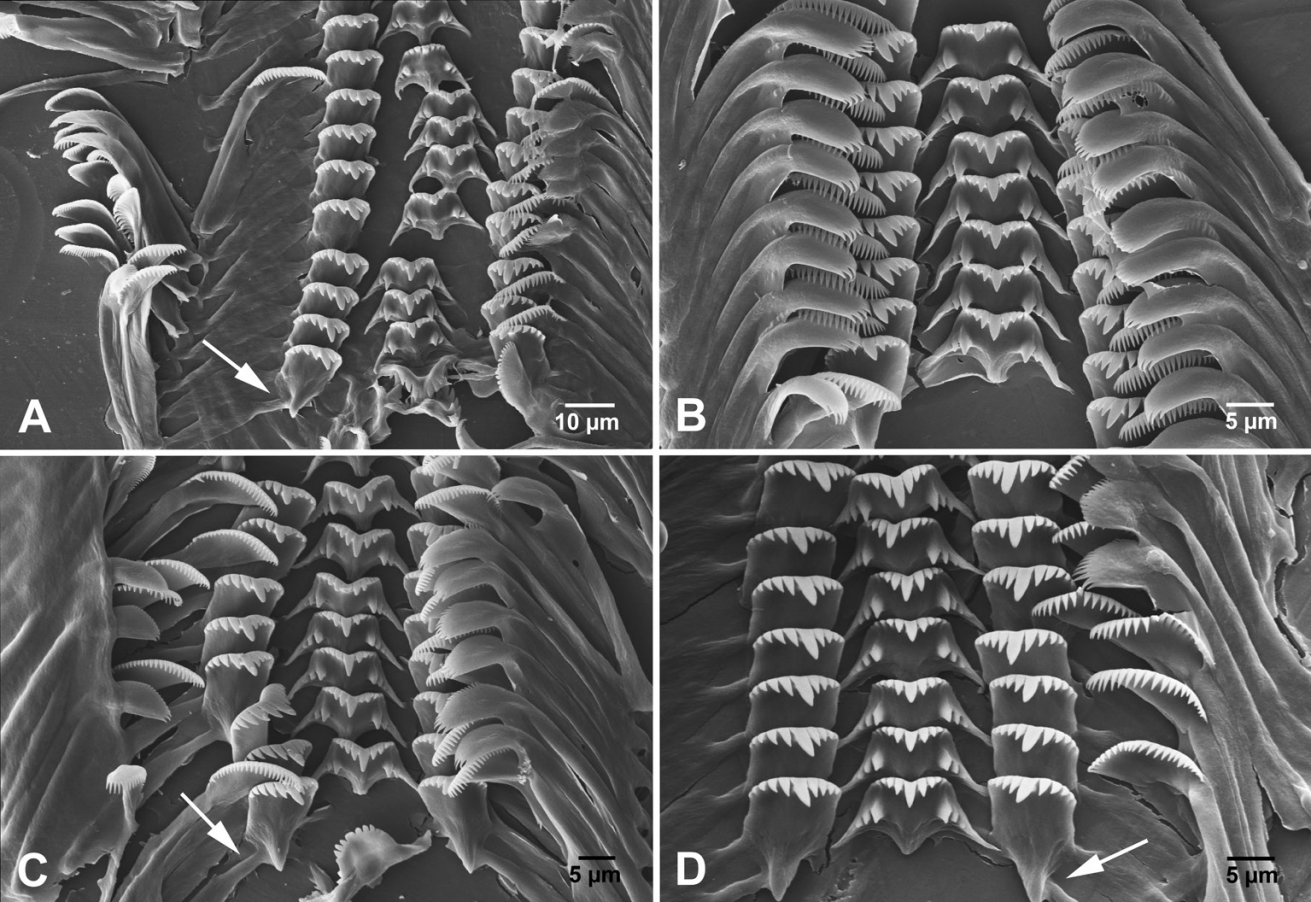
Radula. **A** *Hemistomia andreae* sp. n. **B** *Leiorhagium adioincola* sp. n. **C** *Leiorhagium aremuum* sp. n. **D** *Leiorhagium neteae* sp. n. Arrows indicate membranous junction of flank and face of lateral teeth typical for most Pacific tateid genera (partly dissolved in **A** and **D**).

Radula formula (*N*=3) (Fig. 6A): R (rhachis or central tooth): 3 1 3/2 2, L (lateral tooth): 3 1 5, M_1_ (inner marginal tooth): 21–25, M_2_ (outer marginal tooth): 27–32; stomach without caecum; rectum close to pallial oviduct in females and to prostate in males.

##### Female genitalia.

**Figure 7.**
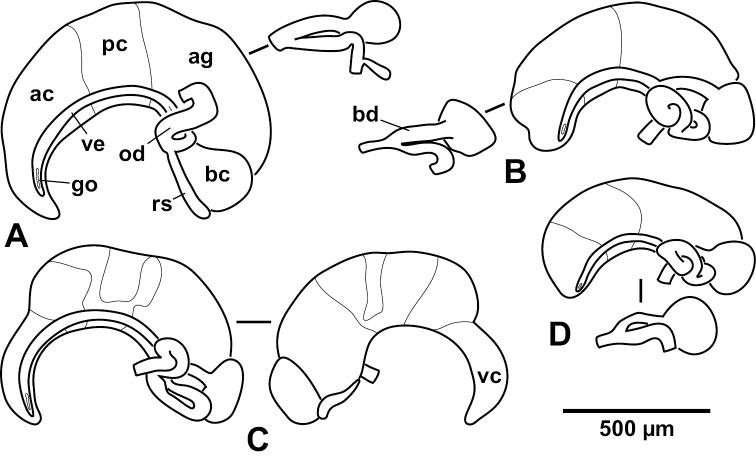
Female genitalia. **A** *Hemistomia andreae* sp. n. **B** *Leiorhagium adioincola* sp. n. **C** *Leiorhagium aremuum* sp. n. **D** *Leiorhagium neteae* sp. n. ac anterior capsule gland, ag albumen gland, bc bursa copulatrix, bd bursal duct, go genital opening, od oviduct, pc posterior capsule gland, rs receptaculum seminis, vc vestibular capsule gland, ve ventral channel.

Ovary without lobes, proximal end c. 1.25 whorls below apex, comprising 0.25–0.5 whorls, eventually reaching stomach; anterior capsule gland yellow-orange, posterior capsule gland opaque-white, albumen gland milky-white; proximal loop of renal oviduct upright comprising 180°, distal loop short; bursa copulatrix pear-shaped, reaching only slightly behind albumen gland; bursal duct long, entering anterior; seminal receptacle on ventral edge of and as long as bursa (*N*=3) (Fig. 7A).

##### Male genitalia.

**Figure 8.**
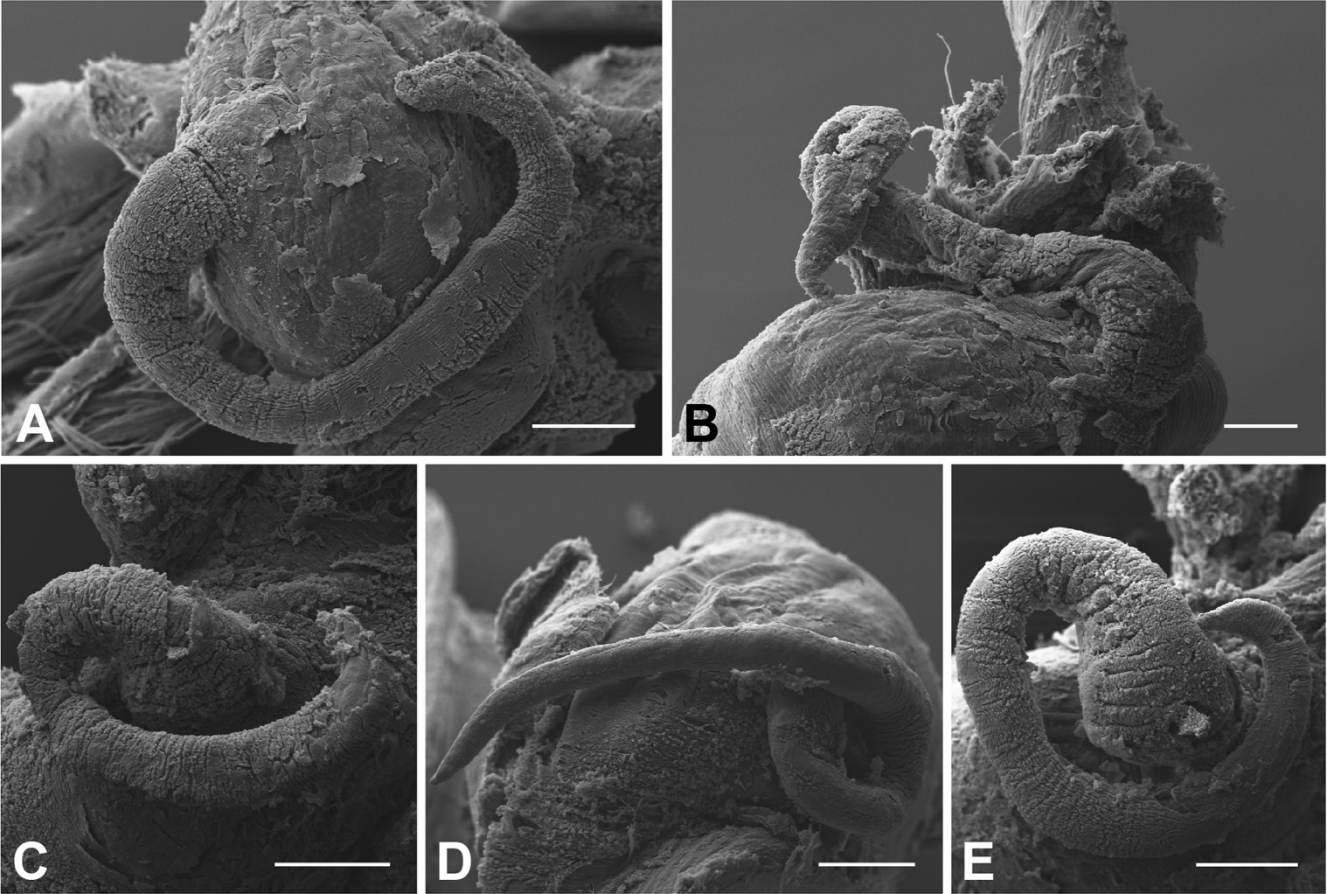
Penis. **A, B** *Hemistomia andreae* sp. n. **C** *Leiorhagium adioincola* sp. n. **D** *Leiorhagium aremuum* sp. n. **E** *Leiorhagium neteae* sp. n. Scale bars = 100 µm.

Proximal end of lobate testis 1–1.25 whorls below apex, comprising 0.75 whorls, covering proximal end of stomach; vesicula seminalis arising from anterior third of testis; penis fairly delicate with blunt end (*N*=2) (Fig. 8A,B).

##### Remarks.

This is *Hemistomia* sp. n. 1 of Zielske and Haase (2015). Both *Hemistomia andreae* sp. n. and *Hemistomia cockerelli* do have the columellar fold in the body whorl assumed to be unique in *Hemistomia nyo* by Haase and Bouchet (1998). *Hemistomia andreae* sp. n. is only known from the type locality.

#### Genus Leiorhagium

Taxon classificationAnimaliaLittorinimorphaTateidae

Haase & Bouchet, 1998

##### Type species.

*Leiorhagium orokau* Haase & Bouchet, 1998

#### Leiorhagium adioincola sp. n.

Taxon classificationAnimaliaLittorinimorphaTateidae

http://zoobank.org/CCC4F863-76C3-44C2-A4AA-CE9DE0B726AB

##### Type material.

Holotype MNHN IM 2000-27860; paratypes MNHN IM 2000-27861 (29), NHMW 110182 (5).

##### Type locality.

NeCa 49, Poya: Massif d’Adio, stream flowing into Grotte d’Adio, open secondary forest, 21°15'24.4"S, 165°14'46.4"E, 29 May 2012.

##### Other material.

NeCa 43, Poya: small stream on W-side of road between Nétéa and Goipin, on forest edge, 21°16'06.0"S, 165°14'32.0"E, 28 May 2012, MNHN-IM-2012-36075 (23), NHMW 110183 (10).

##### Etymology.

Adioincola is composed of the name of the area of Adio and the Latin noun incola meaning inhabitant, and thus refers to the type locality of the new species.

##### Diagnosis.

*Leiorhagium adioincola* sp. n. is very similar to *Leiorhagium kavuneva* and *Leiorhagium clandestinum* sp. n. The former pair differs in penial shape, slender vs. basally broad with long terminal filament. *Leiorhagium adioincola* sp. n. tends to have fewer radular denticles than *Leiorhagium kavuneva*. Genetically, these species differed on average at 9.65% of the positions of COI. Due to the lack of anatomical data, both new species can only be distinguished genetically. Their COI sequences differed on average by 9.5% (p-distance).

##### Shell.

Pupiform, 1.8 times higher than wide, 4.125-4.75 whorls, without colour, transparent; protoconch faintly pitted with c. 1 whorl; palatal denticle a small droplet 1/8 whorl behind outer lip; aperture as high as wide (Figs 2B, 3C, D, 4C, D).

##### Operculum.

Elongate-ellipsoidal, paucisprial, nucleus submarginal, orange, usually two non-calcareous white pegs, eventually accompanied by a small third one (*N*=3) (Fig. 5C,D).

##### External features.

Epidermis without pigment, eyes black.

##### Mantle cavity.

Ctenidium with 18-19 (3 males) or 21–24 (2 females) filaments; osphradium kidney-shaped, behind middle of ctenidium.

##### Digestive system.

Radula formula (*N*=3) (Fig. 6B): R: 4 1 4/2 2, L: 4-5 1 6, M_1_: 22-27, M_2_: 21-29; stomach without caecum; rectum close to pallial oviduct in females and to prostate in males.

##### Female genitalia.

Ovary without lobes, proximal end 1.25 whorls below apex, comprising 0.25-0.5 whorls, eventually reaching stomach; anterior capsule gland yellow-orange, posterior capsule gland opaque-white, albumen gland milky-white; proximal loop of renal oviduct bent forward, distal loop short; bursa copulatrix almost cubical, reaching behind albumen gland; bursal duct long, entering anterior; no seminal receptacle (*N*=2) (Fig. 7B).

##### Male genitalia.

Proximal end of lobate testis 1.25–1.5 whorls below apex, comprising 0.5-0.75 whorls, covering proximal end of stomach; vesicula seminalis arising from anterior half of testis; penis slender, terminal end occasionally forming short filament (*N*=3) (Fig. 8C).

##### Remarks.

This is *Leiorhagium* sp. n. 4 of Zielske and Haase (2015). *Leiorhagium adioincola* sp. n. occurs in the area between the villages of Nétéa and Goipin including the Massif d’Adio.

#### Leiorhagium aremuum sp. n.

Taxon classificationAnimaliaLittorinimorphaTateidae

http://zoobank.org/3B015791-A03B-48BB-8C1D-1A829588B5E2

##### Type material.

Holotype MNHN IM 2000-27862; paratypes MNHN IM 2000-27863 (28), NHMW 110184 (10).

##### Type locality.

NeCa 33, Moindou: spring-fed stream close to road in Aremu valley, under shrub, 21°35'04.8"S, 165°39'07.5"E, 26 May 2012.

##### Etymology.

The new species is named after the Aremu valley, where it has been discovered.

##### Diagnosis.

*Leiorhagium aremuum* sp. n. is most similar to *Leiorhagium ajie*, which is, however, larger and slightly more slender, lacks the palatal denticle, and has a more massive penis. The prolonged capsule gland is unique among New Caledonian tateids. The COI sequences had a p-distance of 9.4%.

##### Shell.

Broadly pupiform, 1.62 times higher than wide, 3.75-4.25 whorls, without colour, transparent; protoconch faintly pitted with 0.75-0.9 whorls; palatal denticle a small droplet 1/8 whorl behind outer lip; aperture practically as high as wide (Figs 2C, 3E,F, 4E,F).

##### Operculum.

Elongate-ellipsoidal, paucisprial, nucleus submarginal, orange, two non-calcareous white pegs, eventually accompanied by a small third one (*N*=4) (Fig. 5E, F).

##### External features.

Epidermis without pigment, eyes black.

##### Mantle cavity.

Ctenidium with 15-16 (2 males) or 19-20 (2 females) filaments; osphradium elongate, slightly behind middle of ctenidium.

##### Digestive system.

Radula formula (*N*=3) (Fig. 6C): R: 4-5 1 4-5/2-3 2-3, L: 4-5 1 4-6, M_1_: 26-31, M_2_: 20-32; stomach without caecum; rectum close to pallial oviduct in females, with short loop left of prostate in males.

##### Female genitalia.

Ovary without lobes, proximal end 1.25-1.75 whorls below apex, comprising 0.25-0.5 whorls, reaching stomach; capsule gland with long and slender, opaque-white vestibulum, anterior capsule gland yellow-orange, toward posterior capsule gland covered with brown spots, posterior capsule gland opaque-white with a central milky section, albumen gland milky-white; proximal loop of renal oviduct bent forward, distal loop long; bursa copulatrix higher than long, reaching behind albumen gland; bursal duct long, entering anterior; no seminal receptacle (*N*=3) (Fig. 7C).

##### Male genitalia.

Proximal end of lobate testis 1 whorl below apex, comprising c. 0.75 whorls, covering proximal end of stomach; vesicula seminalis arising from distal third of testis; penis very long and slender (*N*=2) (Fig. 8D).

##### Remarks.

This is *Leiorhagium* sp. n. 3 of Zielske and Haase (2015). *Leiorhagium aremuum* sp. n. is only known from the type locality.

#### Leiorhagium clandestinum sp. n.

Taxon classificationAnimaliaLittorinimorphaTateidae

http://zoobank.org/723A9EA1-CBFC-486A-AA37-69728E99AC3A

##### Type material.

Holotype MNHN IM 2000-27865; paratypes MNHN IM 2000-27866 (3).

##### Type locality.

NeCa 30, Moindou: spring along road SW of Katrikoin, under shrub, 21°34'21.6"S, 165°41'02.5"E, 26 May 2012.

##### Etymology.

The Latin adjective clandestinus means clandestine and refers to the new species’ external identity with *Leiorhagium kavuneva*.

##### Diagnosis.

*Leiorhagium clandestinum* sp. n. is most similar to *Leiorhagium adioincola* sp. n. and *Leiorhagium kavuneva*. For the distinction from *Leiorhagium adioincola* sp. n. see above. Due to the lack of anatomical data, *Leiorhagium clandestinum* sp. n. and *Leiorhagium kavuneva* can only be distinguished based on 7.6% average sequence divergence of COI (p-distance).

##### Shell.

Pupiform, 1.86 times higher than wide, 4.25-5 whorls, without colour, transparent; protoconch very faintly pitted with c. 1 whorl; palatal denticle a small droplet 1/8 whorl behind outer lip; aperture as high as wide (Figs 2D, 3G, 4G, H).

##### External features.

Epidermis without pigment, eyes black.

##### Remarks.

This is *Leiorhagium* sp. n. 2 of Zielske and Haase (2015). *Leiorhagium clandestinum* sp. n. is only known from the type locality.

#### Leiorhagium neteae sp. n.

Taxon classificationAnimaliaLittorinimorphaTateidae

http://zoobank.org/7B81AF32-3FDA-49C7-A316-D84B1A5ED324

##### Type material.

Holotype MNHN IM 2000-27867; paratypes MNHN IM 2000-27868 (20).

##### Type locality.

NeCa 44, Poya: stream at side of small road branching off road between Nétéa and Goipin toward the Vallée d’Adio, under shrub close to overgrown garden, 21°14'47.9"S, 165°15'45.0"E, 28 May 2012.

##### Etymology.

The new species is named after the village of Nétéa, which is closely proximal to our collecting locality.

##### Diagnosis.

*Leiorhagium neteae* sp. n. is very similar to *Leiorhagium douii* and *Leiorhagium montfaouense*. In *Leiorhagium neteae* sp. n. the palatal denticle is slightly larger and 1/8 whorl further behind the outher lip. The operculum has only a single denticle compared to 2-3 in *Leiorhagium douii* and *Leiorhagium montfaouense*. The distal loop of the renal oviduct of the new species forms a 270° loop counter-clockwise, whereas in the other two species this part of the oviduct is bent 180° clockwise. The penis of *Leiorhagium neteae* sp. n. is long and slender in contrast to the other species, where it has a broad base and a very long filament.

##### Shell.

Elongate-pupiform, 1.88 times higher than wide, 4.25–5 whorls, without colour, transparent; protoconch faintly pitted with c. 1 whorl; palatal denticle an elongate droplet c. 1/4 whorl behind outer lip; aperture slightly wider than high (Figs 2E, 3H, I, 4I, J).

##### Operculum.

Elongate-ellipsoidal, paucisprial, nucleus submarginal, orange, one non-calcareous white peg (*N*=4) (Fig. 5G, H).

##### External features.

Epidermis without pigment, eyes black.

##### Mantle cavity.

Ctenidium with 15 (1 male) or 19-22 (5 females) filaments; osphradium short-elongate, behind middle of ctenidium.

##### Digestive system.

Radula formula (*N*=4) (Fig. 6D): R: 4 1 4/2-3 2-3, L: 4-5 1 5, M_1_: 20-25, M_2_: 24-27; stomach without caecum; rectum close to pallial oviduct in females, with short loop left of prostate in male.

##### Female genitalia.

Ovary without lobes, proximal end 1.25-1.5 whorls below apex, comprising 0.25-0.5 whorls, not reaching stomach; anterior capsule gland yellow-orange, posterior capsule gland opaque-white, albumen gland milky-white; proximal loop of renal oviduct bent forward, distal loop short; bursa copulatrix globular, reaching slightly behind albumen gland; bursal duct long, entering anterior; no seminal receptacle (Fig. 7D).

##### Male genitalia.

Proximal end of lobate testis 1 whorl below apex, comprising slightly more than 0.5 whorls, covering proximal end of stomach; vesicula seminalis arising approximately in middle of testis; penis very long and slender (*N*=1) (Fig. 8E).

##### Remarks.

This is *Leiorhagium* sp. n. 5 of Zielske and Haase (2015). *Leiorhagium neteae* sp. n. is only known from the type locality.

### Morphometry

**Figure 9.**
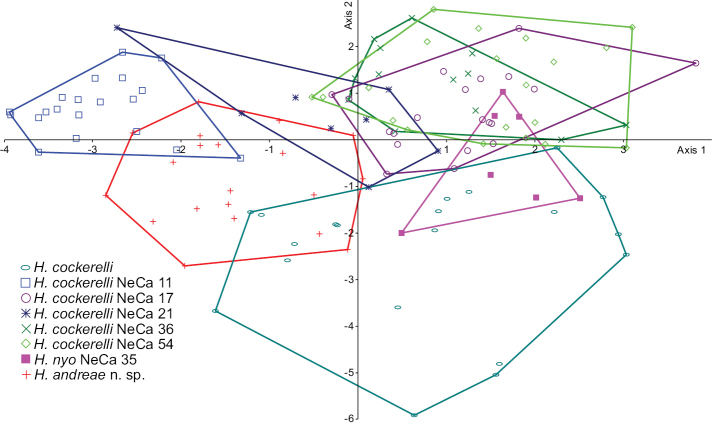
CVA plot for *Hemistomia*. Samples without numbers are paratypes.

The CVA plot (Fig. 9) comparing species of *Hemistomia* shows the high variability of *Hemistomia cockerelli*. The associated MANOVA was highly significant (Wilk’s λ = 0.062, DF_1_ = 35, DF_2_ = 490.4, *F* = 13.16, *p* = < 0.001). Many pairwise comparisons of populations were significant as well (Table 3). *Hemistomia nyo* and *Hemistomia andreae* sp. n. fell within the variation of *Hemistomia cockerelli*. According to the CVA, they were not more different from each other than from populations of *Hemistomia cockerelli*. Assignment and jacknifed assignment tests allocated 80 (62.5%) and 67 (52.3%) of a total of 128 shells to their original sample indicating the considerable overlap of shapes.

**Table 3.** Pairwise morphometric comparisons of *Hemistomia* samples. Hotelling’s T^2^ tests, based on five shell measures; significance assessed after sequential Bonferroni correction; sample sizes are given in Table 2. *
*p* < 0.05; NS, not significant.

	1	2	3	4	5	6	7
1 *Hemistomia andreae*							
2 *Hemistomia cockerelli* Types	*						
3 *Hemistomia cockerelli* NeCa11	*	*					
4 *Hemistomia cockerelli* NeCa17	*	*	*				
5 *Hemistomia cockerelli* NeCa21	NS	*	*	NS			
6 *Hemistomia cockerelli* NeCa36	*	*	*	NS	NS		
7 *Hemistomia cockerelli* NeCa54	*	*	*	NS	*	NS	
8 *Hemistomia nyo* NeCa35	*	*	*	NS	NS	*	NS

**Figure 10.**
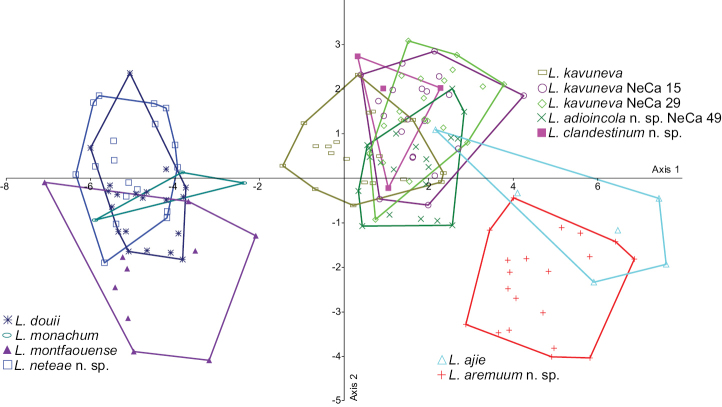
CVA plot for *Leiorhagium*. Samples without numbers are paratypes.

The CVA (Fig. 10) for *Leiorhagium* revealed species clusters with *Leiorhagium adioincola* sp. n. and *Leiorhagium clandestinum* sp. n. overlapping with *Leiorhagium kavuneva* and *Leiorhagium neteae* sp. n. largely grouping with *Leiorhagium douii* and *Leiorhagium monachum*. The MANOVA was again highly significant (Wilk’s λ = 0.009, DF_1_ = 50, DF_2_ = 669.2, *F* = 23.56, *p* = < 0.001), as were most pairwise comparisons (Table 4). Note that comparisons involving *Leiorhagium clandestinum* sp. n. or *Leiorhagium monachum* were less meaningful because of the small sample sizes. Assignment and jacknifed assignment tests performed similar as for *Hemistomia* with only 103 (64.0%) and 88 (54.7%) correctly allocated shells of a total of 161.

**Table 4.** Pairwise morphometric comparisons of *Leiorhagium* samples. Hotelling’s T^2^ tests, based on five shell measures; significance assessed after sequential Bonferroni correction; sample sizes are given in Table 2. *
*p* < 0.05; NS, not significant.

	1	2	3	4	5	6	7	8	9	10
1 *Leiorhagium adioincola* NeCa49										
2 *Leiorhagium aremuum*	*									
3 *Leiorhagium clandestinum*	NS	*								
4 *Leiorhagium neteae*	*	*	*							
5 *Leiorhagium ajie* Types	*	*	NS	*						
6 *Leiorhagium douii* Types	*	*	*	*	*					
7 *Leiorhagium kavuneva* Types	*	*	NS	*	*	*				
8 *Leiorhagium kavuneva* NeCa15B	NS	*	NS	*	*	*	*			
9 *Leiorhagium kavuneva* NeCa29	NS	*	NS	*	*	*	*	NS		
10 *Leiorhagium monachum* Types	*	*	NS	*	NS	NS	*	*	*	
11 *Leiorhagium montfaouense* Types	*	*	*	*	*	NS	*	*	*	NS

### Phylogenetic analysis

**Figure 11.**
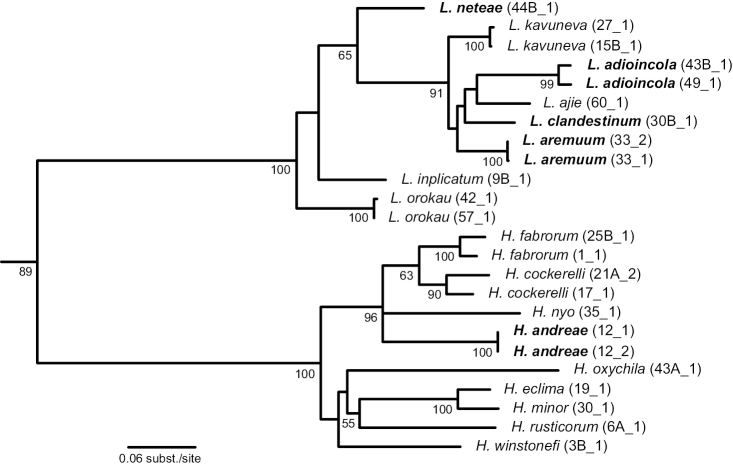
Maximum likelihood phylogram showing bootstrap support when > 50%. Outgroup pruned from tree; new species highlighted by bold face type.

In the phylogenetic analysis (Fig. 11), *Hemistomia* and *Leiorhagium* were sister groups, both with 100% bootstrap support. Within *Leiorhagium*, the elongate-pupiform species *Leiorhagium orokau*, *Leiorhagium inplicatum* and *Leiorhagium neteae* sp. n. were paraphyletic with respect to the more conical-pupiform species, which received a bootstrap support of 91%. Otherwise, relationships among species of *Leiorhagium* were not well supported. All four new species were (phylo)genetically well distinct as indicated by the branch lengths expressing genetic distances. Within *Hemistomia*, the picture was very similar with well differentiated species but otherwise little resolution. Average pairwise uncorrected genetic distances based on the COI-fragment were ≥ 7.4% and are summarized in Table 5.

**Table 5.** Average pairwise uncorrected (p) distances between selected species based on the COI-fragment (in %).

	**1**	**2**		
1 *Hemistomia andreae*				
2 *Hemistomia cockerelli*	8.6			
3 *Hemistomia nyo*	8.8	9.5		
	**1**	**2**	**3**	**4**
1 *Leiorhagium adioincola*				
2 *Leiorhagium ajie*	9.3			
3 *Leiorhagium aremuum*	10.6	9.4		
4 *Leiorhagium clandestinum*	9.5	7.8	7.4	
5 *Leiorhagium kavuneva*	9.7	8.1	8.5	7.6

## Discussion

Our phylogenetic analyses based on DNA sequence data confirmed the suspicion of Haase and Bouchet (1998) that additional cryptic species in this snail fauna will be identified once molecular methods are applied emphasizing the huge morphological variability of certain nominal species. Recent accounts on tateid gastropods from Vanuatu and Fiji (Zielske and Haase 2014a, b) have revealed extensive radiations of morphologically very similar species. However, in contrast to the New Caledonian taxa, the radiations on those archipelagos are comparatively young (Zielske and Haase 2015). Four of the five species described here are hardly distinguishable from known taxa based on measurements despite being genetically well differentiated with even uncorrected distances (see Fregin et al. 2012) of at least 7.4% to their next similar congeners. Whether this means that morphologically similar species occupy similar niches is impossible to tell at this stage because the relationship of shell morphology to habitat has not been investigated among truncatelloidean gastropods except for a few accounts on *Potamopyrgus antipodarum* (Haase 2003, Holomuzki and Biggs 2006, Kistner and Dybdahl 2013). Although ranges overlap or are contiguous, sibling species have not (yet) been encountered in sympatry, i.e. in the same spring or stream.

The new species provide an additional truncatelloid example stressing the importance of an integrative taxonomic approach combining morphological, anatomical and genetic methods (e.g. Haase et al. 2007, Delicado and Ramos 2012). Given the mosaic nature of evolution of these small gastropods with morphologically as well as genetically cryptic species (e.g., Haase et al. 2007, Haase 2008, Zielske et al. 2011, Delicado and Ramos 2012, Liu et al. 2013), we do not adhere to a fairly strict scheme of species identification as advocated elaborately e.g. by Schlick-Steiner et al. (2010). Instead we advocate the approach of Padial and de la Riva (2010) who have a more natural vision of the evolutionary processes potentially involved in speciation. For instance, they acknowledge that the congruence of different character sets, pivotal for taxonomic decisions for Schlick-Steiner et al. (2010), may be plesiomorphic.

Genetic differentiation was an important indicator of species status. Pairwise p-distances > 7.4% are far above any threshold suggested by advocates of barcoding (e.g., Hebert et al. 2003, 2004; Ratnasingham and Hebert 2007). However, again we do not adhere to a strict scheme as there may be no mitochondrial differentiation between good species as well as considerable variation within species of spring snails (e.g. Haase 2008; Zielske et al. 2014a; see also Fregin et al. 2012). That genetic differentiation does reflect species status for the new taxa is also indicated by the comparison of their branch lengths to branch lengths among morphologically well defined species in our phylogenetic analysis.

While conducting our morphometric analyses we appreciated that the measuring methods applied for the material described previously (Haase and Bouchet 1998) and for this account are incompatible. Obviously, using an ocular micrometer fitted to a dissecting microscope produced inaccurate data, although the measurements were quite consistent judging from the fairly low coefficients of variation, which were of a similar order of magnitude as those computed for the present data. Therefore, we had to re-measure the old samples used in our comparisons.

Another methodological problem almost expectedly occurred in the field. All collections made for our previous monograph (Haase and Bouchet 1998) were geo-referenced from maps. This proved to be fairly inaccurate when we tried to relocate sites in 2012 guided by GPS. Additional difficulties arose from recent road development and land-use changes. Many villages are now accessible on much broader roads than 20 years ago. Construction has obviously destroyed small road-side springs and seepages and changed the course of streams. Other sites were destroyed by extensive fires affecting entire valleys or hills. *Crosseana melanosoma*, in our analysis part of the outgroup, used to be common when first collected in 1992. Now we found only a few specimens. It remains to be seen whether there are other (extant) populations in the unexplored hinterland of Boyen. In contrast, *Hemistomia yalayu*, collected in a few seepages on Col d’Amoss in the far Northeast in 1989, is now probably extinct. The entire area has lost its primary vegetation. Today, the fire resistant niaouli (*Melaleuca quinquenervia*) and shrubland are dominating and streams harbor a very depauperate fauna.

Four of the five new species were found in single sites and the fifth was found at only two sites. Considering the vulnerability of small habitats like springs and the rapid anthropogenic development and changes on New Caledonia just outlined immediately raises concern regarding the chances of long-term survival of these species (see also Haase et al. 2010). Most sites we surveyed were rather easily accessible, close to roads, so that one can assume that there are other populations deeper in the forests or forest remnants. Nevertheless, given that the area of occupancy of each species is certainly less than 10 km^2^, that ranges of spring snails are almost naturally severely fragmented, and the rapidly progressing change of land cover, areas of occupancy as well as habitat, hence the numbers of populations will decline. Therefore, all five species and probably the majority of New Caledonian tateids qualify as critically endangered according to the criteria (CE, B2,a,II-IV) of the International Union for Conservation of Nature (IUCN 2012).

## Supplementary Material

XML Treatment for Hemistomia

XML Treatment for Hemistomia andreae

XML Treatment for Leiorhagium

XML Treatment for Leiorhagium adioincola

XML Treatment for Leiorhagium aremuum

XML Treatment for Leiorhagium clandestinum

XML Treatment for Leiorhagium neteae
